# Investigation of *Fusobacterium Nucleatum* in saliva and colorectal mucosa: a pilot study

**DOI:** 10.1038/s41598-022-09587-x

**Published:** 2022-04-04

**Authors:** Amal Idrissi Janati, Igor Karp, Daniel Von Renteln, Mickael Bouin, Younan Liu, Simon D. Tran, Elham Emami

**Affiliations:** 1 Faculty of Dentistry, Université de Montréal, Montreal, QC Canada; 2 Department of Epidemiology and Biostatistics, Schulich School of Medicine and Dentistry, University of Western Ontario, London, ON Canada; 3 Department of Gastroenterology, University of Montreal Hospital Centre, Montreal, QC Canada; 4 McGill Craniofacial Tissue Engineering and Stem Cells Laboratory, Faculty of Dentistry, McGill University, Montreal, QC Canada; 5 Faculty of Dental Medicine and Oral Health Sciences, McGill University, 2001 McGill College Avenue, Suite 500, Montreal, QC H3A 1G1 Canada

**Keywords:** Cancer, Microbiology, Gastroenterology

## Abstract

As evidence has been linking the oral bacterium *Fusobacterium nucleatum (F. nucleatum)* to colorectal tumorigenesis*,* we aimed to produce preliminary data on the expression of *F. nucleatum* in both oral and colorectal body sites in cases diagnosed with colorectal neoplasms (CRN) and CRN-free controls. We conducted a pilot hospital-based case–control study among patients who underwent colonoscopy examination. Saliva samples and biopsies from healthy colon mucosa from CRN cases and CRN-free controls, and from tumors in cases, were collected, as well as data on periodontal condition and potential CRN risk factors. A total of 22 CRN cases and 21 CRN-free controls participated in this study, with a total of 135 biospecimens collected and analyzed by qPCR for detection and quantification of *F. nucleatum*. The detection rate of *F. nucleatum* was 95% in saliva samples and 18% in colorectal mucosa specimens. The median (95% CI) salivary *F. nucleatum* level was 0.35 (0.15–0.82) and 0.12 (0.05–0.65) in case and control groups, respectively, with a Spearman correlation of 0.64 (95% CI 0.2–0.94) between *F. nucleatum* level in saliva and healthy colorectal mucosa in controls. Our study results support the need for and the feasibility of further studies that aim to investigate the association between oral and colorectal levels of *F. nucleatum* in CRN cases and controls.

**Clinical Relevance:** Considering the current evidence linking *F. nucleatum* to colorectal carcinogenesis, investigating the role of oral *F. nucleatum* expression in its colorectal enrichment is crucial for colorectal cancer screening and prevention avenues.

## Introduction

Colorectal cancer (CRC) is the third most commonly diagnosed cancer and the second cause of death from cancer worldwide, with over 1,900,000 newly diagnosed cases and over 900,000 deaths in 2020^1^. Most CRCs arise from adenomatous polyps, which can eventually degenerate into invasive carcinomas^2^. The potential for malignant progress depends on the histologic pattern of growth (with villous pattern being an adverse indicator), size, multiplicity of polyps, and high-grade dysplasia status^3^. A small proportion of CRCs develop under the alternative “serrated pathway”, from serrated polyps frequently located in the proximal colon, and are linked to the Microsatellite Instability phenotype, resulting from a deficiency of the DNA repair system^2^.

Over the last decade, many studies have reported an enrichment of *Fusobacterium nucleatum* (*F. nucleatum)* in colorectal tissues and stools collected from subjects diagnosed with cancerous and even precancerous colorectal lesions^4–6^. The involvement of *F. nucleatum* in early colorectal carcinogenesis stages has been suggested by studies that identified the bacterium in colorectal adenomas, with a gradual enrichment of the colon with *F. nucleatum* in parallel to the adenoma-carcinoma sequence^7–9^. Moreover, the bacterium was identified with two virulence factors promoting colorectal carcinogenesis. The first factor is FadA, an adhesin that allows *F. nucleatum* to invade human epithelial cells, activate β-catenin signaling, induce expression of the oncogenic gene, and promote the growth of colorectal tumor cells^10^. The second factor is a self-transporting protein Fap2, which inhibits the activity of immune cells and thus potentiates the progression of CRC^11^.

*F. nucleatum* is also among the dominant species of the oral cavity^12,13^ and plays an essential role in the formation of dental plaque. It also promotes the colonization and invasion of tooth surfaces by other pathogenic species, which in turn stimulates the recruitment and activation of local immune cells, resulting in destruction of tooth-supporting tissues and progression of periodontitis^14,15^. *F. nucleatum* is abundant in salivary samples from patients with gingivitis and chronic periodontitis^16^.

It has been suggested that gut enrichment with *F. nucleatum* is sourced intra-individually from the oral cavity^17–19^, in the presence of periodontal sites, which may explain the association of periodontal disease (PD) with CRC and colorectal adenomas^20–23^. However, the hypothesis of an intra-individual oral source of gut enrichment with *F. nucleatum* still needs to be tested, which would require epidemiologic data on paired measures of both oral and gut *F. nucleatum* levels in subjects diagnosed with colorectal neoplasms (CRN) and in CRN-free controls. To date, few studies have investigated *F. nucleatum* in saliva from subjects with CRN and CRN-free controls^17,18,24–29^, only two studies have investigated paired saliva and colorectal specimens in these groups^24,28^, and none has explored the link between paired oral and colorectal levels of *F. nucleatum* in subjects with CRN and CRN-free controls (see Table 1 for the studies’ summary).

**Table 1:** Summary of studies that investigated *Fusobacterium nucleatum* in saliva and/or colorectal mucosa or stool in colorectal cancer (CRC) cases and controls (C).

Author -year	Country	No. of CRC cases/C	Exclusion if previous ATB use (period)	Specimen type	Specimen collection time	Collection kits and storage conditions	Bacterial analysis method	*F. nucleatum* detection and level quantification outcomes
Russo et al. 2017	Italy	10/10	Yes (last 3 months)	Unstimulated saliva	1 day before surgery	Sterile tube, − 80 °C	Next generation sequencing & qPCR	***F. nucleatum* level:** In saliva: No significant difference between CRC cases and C In stool: No significant difference between CRC cases and C Saliva **Vs** stool: higher abundance in saliva than in stool, in CRC (p < 0.01) and in C (p < 0.002)
				Stool				
				Fresh tumor mucosa (in CRC cases)	During surgery	0.9% NaCl solution, − 80 °C		
Guven et al. 2019	Turkey	71/77	Yes (last 3 months)	Saliva	Before cancer treatments	Centrifuge tube, − 20 °C	qPCR	***F. nucleatum* detection:** CRC cases: 97.2% **Vs** C: 96.1%; p > 0.99 ***F. nucleatum* level: **(in Log10 copies/ml) CRC cases: 6.89 ± 1.07 **Vs** C: 6.35 ± 0.78; p = 0.001
Komiya et al. 2019	Japan	14/0 (No C)	Yes (last month)	Saliva	Before/after colonoscopy	Sterile tubes, anaerobic conditions	PCR (conventional)	***F. nucleatum* detection:** Saliva: 100% Tumor mucosa: 57% Saliva and tumor mucosa: 43% (*F. nucleatum* identical strain in 75% of patients with both saliva and tumor positive to *F. nucleatum*)
				Tumor mucosa	During colonoscopy			
Kato et al. 2016	USA	68 /122	–-	Oral rinse	–-	Commercial mouthwash (15% alcohol), − 80 °C	16SrRNA gene sequencing	**Dominant Phyla:** Fusobacteria was not dominant, only 3.7% of all sequences No association between *F. nucleatum* and CRC
Abed et al. 2020	Israel	7/0 (No C)	No: ATB was taken just before surgery	Saliva	1 day before surgery, or just after colonoscopy	Sterile tubes, anaerobic conditions	PCR (conventional)	***F. nucleatum* detection:** Saliva: 100% Tumor mucosa: 100%
				Tumor mucosa	45 min after resection			
		3/0 (No C)	Yes (just before surgery)	Saliva	1 day before surgery, or just after colonoscopy	Sterile tubes, anaerobic conditions	Whole genome sequencing	Great similarity between *F. nucleatum* strains in saliva and tumor in each subject
				Tumor mucosa	45 min after resection			
Kageyama et al. 2019	Japan	24/118	Yes (last month)	Stimulated saliva	Before cancer therapy	Sterile tube, − 80 °C	16SrRNA gene sequencing	**Differentially abundant OTUs:** OTUs corresponding to *F. nucleatum* were not the most abundant bacteria OTUs in CRC
Yang et al. 2019	USA	231 / 462	Yes (last week)	Oral rinse	–	Commercial mouthwash, − 80 °C	16S rRNA gene sequencing	***F. nucleatum* detection:** CRC cases: 99.6% **Vs** C: 99.6% (p = 1)
Flemer et al. 2017	Ireland	99/103 + (32 polyp patients)	Yes (last month) (ATB during surgery time)	Oral swabs (45 CRC& 25 C)	–	− 80 °C	16S rRNA gene amplicon sequencing	***F. nucleatum* abundance:** *Fusobacterium* less abundant in oral swabs of CRC cases compared to C
				Stool	Before colonoscopy	− 80 °C		
				Colorectal and tumor mucosa	During surgery or colonoscopy	RNA later at 4 °C for 12 h, then at − 20 °C		

*No.* Number, *CRC* Colorectal cancer, *C* Controls, *F. nucleatum Fusobacterium nucleatum*, *ATB* Antibiotic, *OTU* Operational taxonomic unit, – Not reported.

## Objectives

This pilot study aimed to generate preliminary data on detection and quantification of *F. nucleatum* in both saliva and colorectal mucosa in subjects diagnosed with CRN and CRN-free controls. Ultimately, these preliminary data can help in designing a subsequent large epidemiological study investigating *F. nucleatum* in both oral and colorectal sites concomitantly in subjects diagnosed with CRN and CRN-free controls.

## Methodology

We carried out a pilot hospital-based case–control study in the setting of University of Montreal Hospital Center in Montreal, Quebec, Canada. Participants were consecutive patients who underwent colonoscopy in the gastroenterology department between February 2018 and November 2019. Specifically, we identified patients who were scheduled for colonoscopy exam as part of CRC screening, or as a CRC diagnostic test upon recent change in bowel habits, rectal bleeding, unexplained iron deficiency anemia, or a positive Fecal Immunochemical Test. Patients with advanced colorectal adenoma or CRC were also identified among patients scheduled for an endoscopic mucosal resection technique, after they were diagnosed with polyps suspected of being neoplastic, based on a recent medical imaging or colonoscopy. Endoscopic mucosal resection is indicated for resection of the carpet-type adenomatous colonic polyp, and superficial early colorectal cancers that are well and/or moderately differentiated and limited to the mucosa^30^.

The study inclusion criteria were: (1) aged 40–80 years; (2) resident of Montreal metropolitan area; (3) speaking French and/or English; (4) no prior diagnosis of cancer; (5) no history of hereditary colorectal disease; (6) no history of inflammatory bowel disease; and (7) no history of treatment with antibiotics within the past 3 months.

Patients with histologically confirmed advanced colorectal adenoma or CRC were included in the “case” group of CRN. Advanced colorectal adenoma refers to adenomas with high risk of malignant transformation, which is defined by one or more of the following criteria being met: 3–10 adenomas; high-grade dysplasia; tubulovillous or villous appearance; adenoma > 1 cm in diameter; serrated sessile adenomas^31^. Patients whose colonoscopy did not result in the diagnosis of CRC, colorectal advanced adenoma, or inflammatory bowel disease were included in the ‘control’ group.

Eligible patients who agreed to participate in the study were invited to complete a multi-item study questionnaire, provide a saliva sample, and provide consent for biopsy collection during colonoscopy examination. The study was approved by the University of Montreal Hospital Centre Research Ethics Committee under the number: 2017-7068, CE 16.375—MJB, and all study participants signed the study consent before undergoing their colonoscopy. Participants were confirmed for eligibility only after colonoscopy examination. Participant status (case or control) was confirmed by histological investigation.

### Data collection

Participants were administered a multi-item study-questionnaire that had been used by the research team in a previous population-based case–control study, COLDENT study, investigating the association between PD and sporadic CRC^32^. The questionnaire included different sections on sociodemographic and medical history information, cigarette smoking, anthropometric measures, non-steroidal anti-inflammatory drugs use, oral health, dietary habits, and total physical activity^32–36^. A life-course approach was used to document cumulative long-term history regarding cigarette smoking, specific dietary habits, and physical activity.

Thus, collected data enabled the description of study participants regarding sociodemographic characteristics, periodontal health status, as well as potential risk factors of CRN/CRC, namely age, gender, education attainment, income, body mass index, history of type II diabetes, history of CRC in first-degree relatives, history of regular use of non-steroidal anti-inflammatory drugs, lifetime cumulative cigarette smoking, consumption of red meats, processed meats, and total alcoholic drinks since early adulthood, as well as lifetime total physical activity score. Positive history of PD was defined as self-reported PD with bone loss, a previous professional diagnosis or treatment of PD, or history of clinical symptoms and complications of the disease, such as frequent gum bleeding, tooth mobility, or tooth loss because of PD or tooth mobility^32^.

### Collection of biospecimens

In preparation to colonoscopy examination, all participants received the protocol for conventional bowel preparation, which consists of a diet restricted in residue for 2 days, followed by a strict liquid diet and laxatives (Bi-Peglyte^®^ and Dulcolax 5 mg^®^) in the day before colonoscopy. During colonoscopy, biopsies were taken from healthy mucosa in cases and controls, and from polyps (or tumors) in cases. Given the differences in gut microbial composition between proximal and distal colon sites, biopsies of healthy mucosa were separately collected from ascending and descending colon. Biopsies of polyps were taken from freshly excised polyps before they were sent for histopathology analysis. If a clinical decision was made during colonoscopy to delay a polyp removal and take biopsies for histopathology analysis (when a malignant lesion is suspected), an extra-biopsy was then taken for the present study analysis. All biopsies were collected in physiological solution (Nacl 0.9%), then immediately transferred to empty sterile containers.

Unstimulated saliva was collected from participants the day of colonoscopy, or a few days later (at the time of interview), by spitting in a commercial collection kit for DNA stabilization (DNAGenotek (OMNI gene•ORAL | OM-501 kit^®^). Participants were warned not to eat, drink, smoke, or chew gum for 30 min before saliva collection. Mucosa and saliva specimens were immediately stored at − 80 °C until analysis.

### Bacterial DNA extraction and quantitative polymerase chain reaction (qPCR)

Genomic DNA was isolated from saliva and colon tissue samples using the QIAamp DNA Mini Kit (Cat#51304, Qiagen, USA) and procedures were done according to the manufacturer’s instructions. DNA content was quantified using the Bio-Rad SmartSpec 3000 Spectrophotometer (Bio-Rad, 170–2501, USA). DNA sequences of TaqMan primer and probe used to detect 16S ribosomal RNA gene of *F. nucleatum* were similar to those described by Mima et al.^37^: F. nucleatum forward primer, 5′-CAACCATTACTTTAACTCTACCATGTTCA-3′; F. nucleatum reverse primer, 5′-GTTGACTTTACAGAAGGAGATTATGTAAAAATC-3′; F. nucleatum FAM probe, 5′-GTTGACTTTACAGAAGGAGATTA-3′. For human colon tissue, SLCO2A1 was used as endogenous control gene (Hs01114926_m1, FisherThermo Scientific, USA). For human saliva, the MEFE gene (Ba042114926-s1, FisherThermo Scientific, USA) was used as reference gene. A total of 80 ng DNA was used in qPCR reaction and the total reaction volume was 10 ul. Amplification and detection of DNA was performed with the StepOnePlus Real-Time PCR Systems (Applied Biosystems, USA), using the following reaction conditions: 10 min at 95 °C, 40 cycles of 15 s at 95 °C, and 1 min at 60 °C. For quality control, DNA of *F. nucleatum* strain ATCC 25,586 was used as a positive control. No DNA loading and Diethyl pyrocarbonate (DEPC) treated water were used as negative controls. *F. nucleatum* positivity was defined as a detectable level of *F. nucleatum* DNA within 40 PCR cycles, and *F*. *nucleatum* negativity was defined as an undetectable level with a proper amplification of human reference gene SLCO2A1. The bacterium level relative quantification is automatically provided by StepOne Plus Realtime PCR Systems (Applied Biosystems, USA) as 2^−ΔCq^ value, with ΔCq = average Cq value of *F*. *nucleatum* − average Cq value of total bacteria or of the reference gene.

### Statistical analysis

Since this is a pilot study, the statistical analysis performed was purely exploratory, in order to help future studies in study-design decisions, including sample-size calculations.

The distributions of relevant characteristics concerning CRN risk factors in the case and control series were presented with mean and standard deviation, or median and inter-quartile range (when data seemed non-normally distributed) for continuous variables, and percentage for categorical variables. Based on data from qPCR analysis of study specimens*,* we calculated both frequencies of positive detection of *F. nucleatum* and medians of 2^−ΔCq^ with their corresponding 95% confidence intervals (CI), in each group (cases and controls) and each specimen type (saliva, colorectal mucosa). Also, coefficients of Spearman correlation between salivary and colorectal *F. nucleatum* levels, as well as between *F. nucleatum* levels in heathy mucosa of both the ascending and descending colon were presented with their corresponding 95% CIs. IBM SPSS Statistics version 26 was used for statistical analysis.

### Ethical approval

The study was approved by the University of Montreal Hospital Centre Research Ethics Committee, and we certify that the study was performed in accordance with the ethical standards as laid down in the 1964 Declaration of Helsinki and its later amendments.

### Informed consent

All study participants have provided an informed consent to participate in the study.

## Results

A total of 75 potentially eligible participants were solicited, of whom 20 did not meet the study eligibility criteria, and 12 refused to participate. Therefore 43 patients participated to this pilot study, including 22 cases of CRN and 21 CRN-free controls.

All participants, except one case, completed the study questionnaire. Distributions of cases and controls according to sociodemographic and other relevant characteristics are presented in Table 2. In general, case and control groups were similar regarding education attainment (mostly college or university), family history of CRC, and history of diabetes. However, cases were mostly males (81%), slightly older, and less regular users of non-steroidal anti-inflammatory drugs than controls. Also, although the frequency of cigarette smoking was similar in the two groups, the median number of packs-years among smokers in the case group was much higher than for smokers in the control group. Patients in the control group consumed more red and processed meats but fewer alcoholic drinks than cases. Ten participants (7 cases and 3 controls) had a positive history of PD.

**Table 2:** Sociodemographic characteristics and potential colorectal neoplasm risk factors in study participants.

Characteristic	Cases, n = 22	Controls, n = 21
Age, years Mean (SD)	63.9 (9.6)	60.4 (9.1)
**Gender, n (%)**		
Male	18 (82)	10 (48)
**Canadian born**		
Yes, n (%)	16 (73)	18 (86)
**Native tongue**		
French, n (%)	15 (68)	20 (95)
**Education attainment**		
College or university, n (%)	15 (68)	15 (71)
**Living alone**		
Yes, n (%)	5 (23)	11 (52)
**BMI, kg ∕m**^**2**^		
Mean (SD)	27.7 (6)	26.2 (4)
**Family history of CRC**		
Yes, n (%)	4 (18)	2 (10)
**Regular use of NSAIDs**		
Yes, n (%)	3 (14)	9 (43)
**Diabetes**		
Yes, n (%)	3 (14)	3 (14)
**Periodontal disease**		
Yes, n (%)	7 (32)	3 (14)
**Personal income (CAD$ per year)**		
Median (IQR)	35 000 (40 000)	45 000 (80 000)
**History of smoking**		
Positive, n (%)	14 (64)	14 (67)
**Cigarette smoking, packs-years**		
Median (IQR)	22.5 (33.9)	14.4 (27)
**Lifetime average daily total alcoholic drinks**^**a**^		
Median (IQR)	1 (1.62)	0.8 (1.2)
Lifetime average weekly servings^b^ of:		
Red meats Processed meats Median (IQR)	2.1 (3.1) 1.5 (2.1)	3.2 (5.6) 1.8 (3.3)
Lifetime total physical activity score, MET hour/week/year Median (IQR)	88.7 (95.7)	70.5 (114)

*BMI* Body mass index, *NSAID* Non-steroidal anti-inflammatory drugs, *CAD$* Canadian dollars, *MET* Metabolic equivalent of task; a: one drink including beer (355 ml bottle or can), wine (180 ml), or liquor (150 ml); b: 1 serving of red meats = 180–240 g, 1 serving of processed meats = 55 g; SD: Standard deviation; IQR: Interquartile range.

All cases had undergone polypectomy or endoscopic mucosal resection during colonoscopy, and one polyp was removed in each patient, except for two patients with two polyps removed during the same colonoscopy, bringing the total CRN specimens to 24. Characteristics of the CRN (location, histological type, and size) are presented in Table 3. Fifteen polyps were located in the proximal colon, and 9 in the distal colon. Upon the histopathology report, 15 polyps were conventional adenomas (namely, of the tubular, tubulovillous, or villous histology type), two were serrated sessile adenomas, and two early-stage CRCs. Besides specimens of CRN, biopsy specimens were collected from healthy mucosa in 17 cases (13 from ascending colon and 14 descending colon) and 21 controls (20 from ascending colon and 21 descending). Saliva samples were collected in all participants. Thus, a total of 135 study biospecimens were analyzed by qPCR for detection and quantification of *F. nucleatum* levels.

**Table 3:** Characteristics of colorectal neoplasms in case group.

Cases	Colorectal neoplasms				Paired healthy mucosa: biopsy collection site	
	Colorectal anatomic site (segment)	Histologic type (subtype)	High grade dysplasia	Size^ǂ^ (cm)	Ascending colon	Descending colon
3	Proximal (Caecum)	SA (sessile serrated)		2	X	X
4	Proximal (Transverse)	SA (sessile serrated)		6	X	X
5	Proximal (Ascending)	CA (Tubular)		4	X	X
6	Proximal (Ascending)	CA (Tubular)		16	X	X
7	Proximal (Hepatic flexure)	CA (Tubular)	X	3		
	Proximal (Ascending)	CA (Tubular)		5		
9	Distal (Rectum)	CA (Tubular)		2.5		X
10	Proximal (Ascending)	CA (Tubulovillous)	X	5	X	X
11	Proximal (Caecum)	CA (Tubulovillous)		5	X	X
12	Proximal (Caecum)	CA (Tubulovillous)		4.5		
13	Proximal (Ascending)	CA (Tubulovillous)	X	3.5		
14	Proximal (Caecum)	CA (Tubulovillous)		5	X	X
15	Proximal (Hepatic flexure)	CA (Tubulovillous)		2	X	
16	Distal (Sigmoid)	CA (Tubulovillous)		2.5	X	X
17	Distal (Rectum)	CA (Tubulovillous)	X	10	X	X
18	Distal (Rectum)	CA (Tubulovillous)		2.5		X
19	Distal (Rectum)	CA (Tubulovillous)		5		
	Distal (Recto-sigmoid)	CA (Tubulovillous)		2		
21	Distal (Rectum)	CA (Tubulovillous)	X	7		
22	Proximal (Transverse)	CA (Villous)		4	X	
23	Proximal (Ascending)	CA (Villous)		5	X	
24	Proximal (Caecum)	CA (Villous)	X	5		X
25	Distal (Rectum)	CRC (High-grade intraepithelial epidermoid neoplasia)	X			X
26	Distal (Sigmoid)	CRC (moderately differentiated adenocarcinoma developed on a villous adenoma	X	7		X

*SA* Serrated adenoma, *CA* Conventional adenoma, *CRC* colorectal cancer, *X* applicable.

^ǂ^The largest diameter is reported.

**Figure 1:**
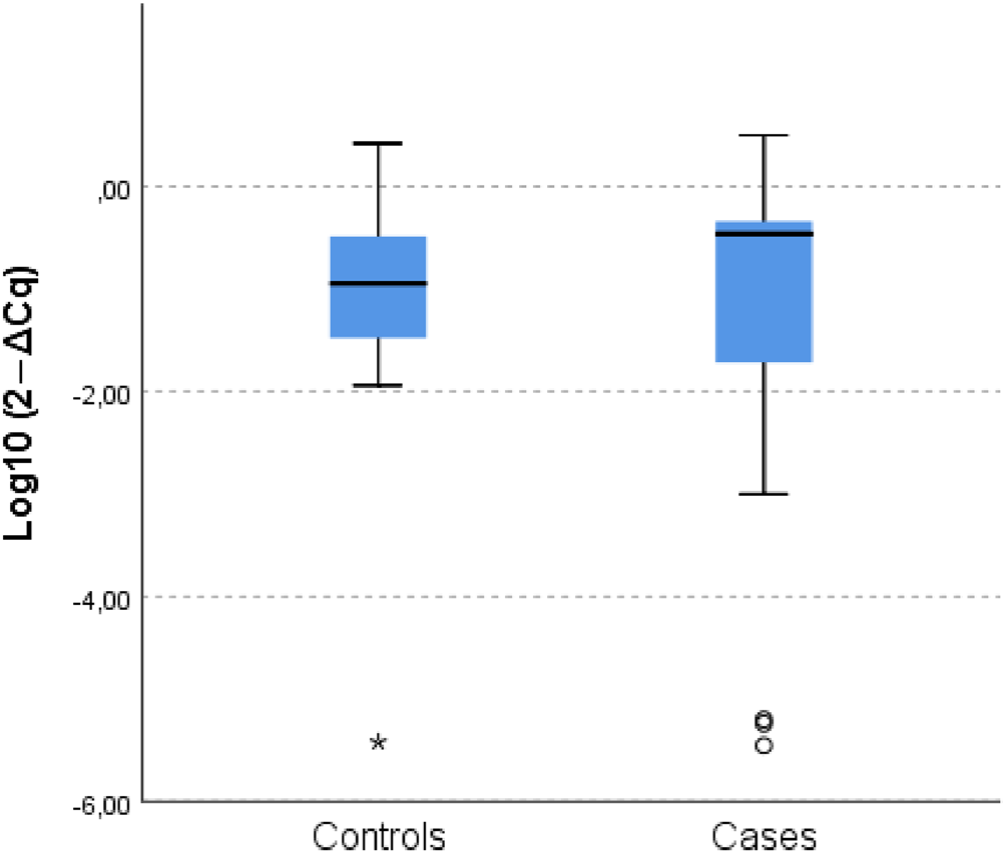
Relative quantification of Fusobacterium nucleatum level in saliva, in case and control groups. Fusobacterium nucleatum level in saliva specimens is measured by qPCR as 2 − ΔCq.

Table 4 shows the *F. nucleatum* detection rate by biospecimen type (saliva, mucosa), in case and control groups. *F. nucleatum* was detected in saliva specimens from almost all cases (21/22) and controls (20/21). *F. nucleatum* levels (measured by qPCR as 2^−ΔCq^) in saliva ranged from barely detectable (0.000004) to 3.17 and 2.65, in cases and controls respectively. The median (95% CI) of salivary *F. nucleatum* level was 0.345 (0.15–0.82) and 0.12 (0.05–0.65) in case and control groups respectively (Fig. 1); and 0.4 (0.13–0.53) in participants with positive history of PD vs 0.14 (0.18–0.73) in participants with negative history of PD.

**Table 4:** Detection frequency of *Fusobacterium nucleatum* by specimen type and participant group.

Specimen type	Case group (n = 22)		Control group (n = 21)	
	Total number of specimens	*F. nucleatum *detected, n	Total number of specimens	*F. nucleatum *detected, n
Saliva	22	21	21	20
Healthy mucosa-ascending colon	13	1	20	9
Healthy mucosa-descending colon	14	1	21	6
Colorectal neoplasms	24	1	NA	NA

*F. nucleatum* *Fusobacterium nucleatum*, *NA* non-applicable.

In colorectal mucosa, *F. nucleatum* was detected in only one case (5%) within both healthy mucosa (from both ascending and descending colon specimens) and polyp, and in 9 controls’ healthy mucosa specimens (ascending and/or descending colon) (43%). The polyp where *F. nucleatum* was detected was a conventional adenoma, tubular subtype, located in the proximal colon.

The level of *F. nucleatum* in controls’ healthy mucosa specimens ranged from 0.116 to 2.02 in the ascending colon, and from 0.045 to 1.2 in the descending colon. *F. nucleatum* level in healthy mucosa from the case detected with *F. nucleatum* was higher than the maximum observed in controls: 2.574 in the ascending colon, 1.143 in the descending colon, and 1.952 in the polyp.

The Spearman correlation coefficient between *F. nucleatum* levels in saliva and mucosa samples was 0.64 (95% CI: 0.2–094). This was calculated for controls only, as in cases only one subject had *F. nucleatum* detected in both saliva and colorectal mucosa specimens. We also explored if there was any correlation between levels of *F. nucleatum* in heathy mucosa of both ascending and descending colon, and the corresponding Spearman correlation coefficient was calculated as 0.68 (95 CI 0.25–0.96).

## Discussion

In this pilot study, we generated preliminary data on detection and quantification of *F. nucleatum* in both saliva and colorectal mucosa from patients diagnosed with CRN and CRN-free controls.

We were able to collect and analyze a total of 135 biospecimens including saliva samples and healthy-colon mucosa biopsies from most cases and controls, and CRN biopsies from all cases.

### F. nucleatum in saliva from CRN cases and CRN-free controls

Analysis with qPCR showed high detection rates of *F. nucleatum* in saliva from both case and control groups, consistent with previous studies^25,29^. *F. nucleatum* is in fact a commensal bacterium of the oral cavity, which explains its more common detection in saliva regardless of disease status. We also found a higher median level of *F. nucleatum* in saliva from the case group than from controls. A recent study in Turkey with a large number of participants (71 CRC cases and 77 controls)^25^, and also using qPCR for microbial saliva analysis, showed a higher mean amount of *F. nucleatum* in CRC group than in control group (6.89 Log10 copies / ml in the case group vs 6.35 in the control group, p = 0.001). However, two other studies that applied 16SrRNA gene sequencing found similar salivary levels of *F. nucleatum* relative abundance in cases and controls. The first study was conducted in the USA^27^, and saliva was collected by oral rinse with a commercial mouthwash, among 68 CRC cases and 122 controls. The second was conducted in Japan^26^, and included unstimulated saliva from 24 CRC cases (and other cancers of the digestive tract) and 118 controls. In both studies, Fusobacterium was not the dominant bacterium.

### F. nucleatum in colorectal mucosa from CRN cases and CRN-free controls

We found a low global detection rate of *F. nucleatum* in colorectal mucosa specimens in the controls, and it was even lower in the cases. At first, this finding might appear contradictory to previous reports finding *F. nucleatum* to be associated with CRN^7–9^. However, when considering the histologic type and the location of CRN in the patients in our study, our results can be seen to be consistent with those previous findings. Mima et al.^38^ analyzed 1,102 colorectal tumors with qPCR, in 13% of which *F. nucleatum* was detected. When analyzing by colorectal tumor site, *F. nucleatum* detection was 15% and 9% in proximal and distal-rectal sites, respectively. Also, in a previous study by Yu et al.^39^, where *F. nucleatum* was investigated in 280 CRNs and 20 healthy mucosa specimens from independent controls using FISH technique, that was further validated in 20 samples by *F. nucleatum*-specific PCR primers, *F. nucleatum* was prevalent in proximal serrated sessile adenomas, but rare in conventional adenomas^39^. According to that study, the frequency of *F. nucleatum* positivity (defined as > 5 visualized probes per field) was 29% in proximal conventional adenomas, 24% in distal conventional adenomas, 79% in serrated sessile adenomas, 90% in proximal CRCs, 42% in distal CRCs, and 20% in healthy mucosa from independent controls. High abundance of invasive *F. nucleatum* (defined as ˃ 20 visualized probes per field) was present in 5.3% of proximal conventional adenomas, 2.4% of distal conventional adenomas, 49% of serrated sessile adenomas, 71% of proximal CRCs, 38% of distal CRCs, and none of the 20 healthy mucosa samples. We point out that the CRNs sampled in our study included 12 proximal conventional adenomas, 6 distal conventional adenomas, 2 serrated sessile adenomas and 2 distal CRCs, and that *F. nucleatum* was detected in a proximal conventional adenoma.

In this pilot study, we noticed that *F. nucleatum* was usually either detected in both subject’s proximal and distal colon sites (ascending and descending colon healthy mucosa specimens), or not detected at all in both colon sites. On the other hand, we noticed that *F. nucleatum* level in the ascending colon moderately correlated with level in the descending colon. This could probably be because some subjects naturally harbor *F. nucleatum* in their gut microbiome, whereas others do not, which can also explain the detection of *F. nucleatum* in healthy mucosa of some controls in many previous studies. We can also think *F. nucleatum* may be associated to the intestinal disorders that led patients in the control group to undergo colonoscopy, and that it may be particularly involved in the serrated neoplasia pathway (where sessile serrated adenomas are precursors to tumors with sporadic microsatellite instability), and less in the conventional adenoma-carcinoma sequence, as suggested by Yu et al.^39^.

### Comparison of F. nucleatum in saliva and in colorectal mucosa within CRN cases and CRN-free controls

Detection rate of *F. nucleatum* in colorectal mucosa was much lower than in saliva, and few subjects had *F. nucleatum* detected in both sites. We found a moderate correlation between *F. nucleatum* level in saliva and healthy proximal colorectal mucosa in controls, but we could not explore this correlation in cases as *F. nucleatum* was detected in colorectal mucosa specimens of only one CRN case. The only data that could serve as comparison to our finding came from two previous studies that investigated *F. nucleatum* in a few samples of saliva and colorectal tumors in the same CRC cases, without control group and without bacterium quantification, as only conventional PCR (non-quantitative) was used^18,28^. *F. nucleatum* was detected less commonly in mucosa samples than in saliva in the first study (in 8/14 tumors and 14/14 saliva)^18^, and in all specimens in the second one (in 10 tumors and 10 saliva)^28^.

In conclusion, concerning the objectives of the pilot study, our study findings provide potentially useful preliminary data on expression of *F. nucleatum* in both oral and colorectal body sites in patients diagnosed with CRN and CRN-free controls. Further studies that aim to assess the association between oral and colorectal levels of *F. nucleatum* in CRN cases are still needed and can draw from our methods and results in making study-design decisions, including the inclusion/exclusion criteria, selection of study participants, data collection instruments and analysis, and sample size calculation. They also should pay attention to both the histologic type and site of CRNs to be included, and optimally focus on proximal location and adenoma of sessile type. To quantify *F. nucleatum* in colorectal healthy mucosa, there may no longer be a need for collection and analysis of two different specimens from both proximal and distal colon sites, as one specimen (preferably from the proximal colon) can be sufficiently informative, especially given that some patients may not consent to provide biopsies from healthy mucosa even if they agree to provide specimens of their tumor, which they know will be excised anyway. Finally, studies should generally plan a quantitative microbial analysis of *F. nucleatum* in saliva specimens: non-quantitative techniques (such as conventional PCR, for example) only assess the presence of the bacterium.

Our preliminary results encourage future research to investigate oral and colorectal enrichment in *F. nucleatum*, in patients with precancerous lesions as well as cancerous lesions at different stages of colorectal malignant transformation, to overcome the difficulty of conducting prospective research on the causal role of the oral bacterium *F. nucleatum* in colorectal carcinogenesis. If the hypothesis of an intra-individual oral origin of the colorectal enrichment in *F. nucleatum* is confirmed, this may have potential impact on colorectal cancer prevention, diagnosis, and treatment. Thus, many studies are investigating the potential of candidate fecal bacteria as biomarkers for early detection of adenomatous polyps and colon cancer^40,41^. Under the same perspective, saliva can be a promising non-invasive screening tool for colorectal adenoma and cancer. Also, and more importantly, investigating the association between the oral and colorectal levels of *F. nucleatum* in colorectal neoplasms patients can advance the understanding of the mechanism(s) underlying the connection between periodontal disease and colorectal cancer, which may involve the translocation of periodontal pathogens to the gut and the release of their pro-oncogen and pro-inflammatory virulence products. Periodontal disease is suggested as a risk factor for periodontal disease^42^, but the mechanisms of the association have yet to be elucidated. Thus, a subsequent, larger epidemiological study on this topic is highly recommended.

## Abbreviations

CRC: Colorectal cancer
CRN: Colorectal neoplasm
*F. **nucleatum*: Fusobacterium nucleatum
PCR: Polymerase chain reaction
PD: Periodontal disease
qPCR: Quantitative polymerase chain reaction
